# Cardiologic Phenotypes and Natural History of FAC

**DOI:** 10.1186/1750-1172-10-S1-I18

**Published:** 2015-11-02

**Authors:** Claudio Rapezzi

**Affiliations:** 1 Department of Experimental, Diagnostic and Specialty Medicine, Alma Mater-University of Bologna, Bologna, Italy

## 

Fifty years ago, the focus on the Val30Met type of the disease, in which neurologic manifestations predominate, led to the widespread notion that hereditary transthyretin-related amyloidosis (ATTR) was essentially a neurologic disease. It is now clear that ATTR is extremely heterogeneous on both genotypic and phenotypic grounds. The clinical spectrum of the disease ranges from an almost exclusive neurologic involvement to strictly cardiac manifestations. This heterogeneity is linked to several factors including specific transthyretin mutations, geographic distribution and endemic vs. non-endemic aggregation type. The existence of exclusively or predominantly cardiac phenotypes makes the recognition of the disease very challenging since it can mimic other more common causes of left ventricular “hypertrophy”. Assessment of such patients should include an active search for possible red flags that can indicate the correct final diagnosis. More in general the clinician must be aware that:

• Cardiac amyloidosis (CA) should be suspected in any patient with heart failure, unexplained increased LV wall thickness and non-increased end systolic left ventricular volume

• In a patient with an initial diagnosis of hypertrophic cardiomyopathy (HCM), look for the infiltrative phenotype hidden beneath the hypertrophic one!

• A distinctive sign of CA is the abnormal ratio between left ventricular thickness and QRS voltages rather than low QRS voltages, alone. The absence of low QRS voltages doesn’t rule out a CA if the context is otherwise fitting and up to 20% of subjects with CA can have electrocardiographic evidence of left ventricular hypertrophy.

• In an elderly man with unexplained concentric left ventricular hypertrophy, especially in the absence of hypertension, always consider the possibility of wtTTR CA!

• Amyloidotic cardiomyopathy in an elderly patient with monoclonal gammopathy is not necessarily related to AL: consider the possibility of wTTR + MGUS

• Longitudinal LV function can be severely depressed despite a normal LVEF and the myocardial contraction fraction is often low suggesting reduced global myocardial shortening.

• Myocardial deformation is reduced in amyloidotic cardiomiopathy but the apex is generally spared

• In CA, Gadolinium distribution at MRI is heterogeneous: subendocardial late gadolinium enhancement (LGE) is not the only diagnostic pattern and the absence of LGE does not exclude CA.

• Bilateral carpal tunnel syndrome in a man with HCM-like phenotype on echo is highly suggestive of TTR-related CA

The natural history of ATTR is only partially known, due to the rarity of the disease and its high genotypic heterogeneity. Patients with ATTR cardiomyopathy have a much more indolent course than those with AL etiology. Progression of neurological disease and heart failure are the leading causes of death. As in AL amyloidosis, in ATTR the presence of cardiac involvement is an incremental risk factor for overall and cardiovascular events. Consequently, non-Val30Met mutations, in which cardiac involvement is more frequent and severe, appear to be associated with a particularly poor prognosis, even after successful OLT . Very little is known about the underlying cause and the incidence of sudden death, which has been reported to be associated with the occurrence of advanced atrioventricular blocks, ventricular tachycardia/fibrillation, or, probably more frequently, electromechanical dissociation.

